# Development of prevalence and incidence of non-tuberculous mycobacteria in German laboratories from 2016 to 2020

**DOI:** 10.1080/22221751.2023.2276342

**Published:** 2023-12-06

**Authors:** Caroline Corbett, Philipp Finger, Marion Heiß-Neumann, Juergen Bohnert, Ines B. Eder, Melanie Eisele, Inna Friesen, Achim J. Kaasch, Jan Kehrmann, Roland Lang, Jürgen Rödel, Susann Roessler, Annika Schmidt, Sophie Schneitler, Daniela Schui, Franziska Schuler, Ludwig Sedlacek, Annerose Serr, Ana-Gabriela Sitaru, Joerg Steinmann, Dirk Wagner, Thomas A. Wichelhaus, the EpiNTM-Group, Sabine Hofmann-Thiel, Harald Hoffmann

**Affiliations:** a Institute of Microbiology and Laboratory Medicine, Department IML red GmbH, WHO - Supranational Tuberculosis Reference Laboratory, Munich-Gauting, Germany; b Department of Pneumology & Infectious Diseases, Asklepios Lung Clinic Munich-Gauting, member of the German Centre for Lung Research; Gauting, Germany; c Friedrich Loeffler-Institute of Medical Microbiology, Greifswald, Germany; d Institute of Medical Microbiology and Virology, University Hospital Leipzig, Leipzig, Germany; e Institut für medizinische Mikrobiologie, Universitätsmedizin Göttingen, Göttingen, Germany; f Labor Berlin - Charité Vivantes GmbH, Berlin, Germany; g Institute of Medical Microbiology and Hospital Hygiene, Medical Faculty, University Hospital Magdeburg, Otto von Guericke University, Magdeburg, Germany; h Institute of Medical Microbiology, University Hospital Essen, University of Duisburg-Essen, Essen, Germany; i Institut für Klinische Mikrobiologie, Immunologie und Hygiene, Universitätsklinikum Erlangen, Erlangen, Germany; j Institute of Medical Microbiology, Jena University Hospital, Friedrich Schiller University, Jena, Germany; k Institut für Medizinische Mikrobiologie und Virologie, Universitätsklinikum Carl Gustav Carus an der Technischen Universität Dresden, Dresden Germany; l Institut für medizinische Mikrobiologie und Hygiene, Tübingen, Germany; m Institute of Medical Microbiology and Hygiene, Saarland University, Homburg/Saar, Germany; n Institute of Pneumology at the University of Cologne, Bethanien Hospital, Clinic for Pneumology and Allergology, Centre of Sleep Medicine and Respiratory Care, Solingen, Germany; o Bioscientia Institut für Medizinische Diagnostik GmbH, Mikrobiologie, Ingelheim, Germany; p Institute of Medical Microbiology, University Hospital, Münster, Germany; q Institute for Medical Microbiology and Hospital Epidemiology, Hannover Medical School, Hannover, Germany; r Institute of Medical Microbiology and Hygiene, University of Freiburg, Freiburg, Germany; s Medizinisches Versogungszentrum Clotten (MVZ) Clotten, Freiburg, Germany; t Institute of Clinical Microbiology, Infectious Diseases and Infection Control, Paracelsus Medical University, Klinikum Nürnberg, Nürnberg, Germany; u Division of Infectious Diseases, Department of Internal Medicine II, Medical Center – University of Freiburg, Faculty of Medicine, University of Freiburg, Freiburg, Germany; v Institute of Medical Microbiology and Infection Control, University Hospital Frankfurt, Frankfurt am Main, Germany; w SYNLAB Gauting, SYNLAB MVZ Dachau GmbH, Munich-Gauting, Germany

**Keywords:** Non-tuberculous mycobacteria, prevalence, incidence, drug susceptibility, NTM-pulmonary disease

## Abstract

Numbers of non-tuberculous mycobacteria (NTM) pulmonary diseases (PD) have been repeatedly reported as increasing over the last decades, particularly in Europe. Sound epidemiological data are however missing for most European regions. This study calculated prevalence and incidence of NTM recovered from patients’ lungs in Germany, the largest Central European country, over a five-year period. It furthermore determined regional particularities of NTM species and results from susceptibility testing. 22 German NTM laboratories provided their mycobacteriological diagnostic data of 11,430 NTM isolates recovered from 5998 pulmonary patients representing 30% of all notified NTM-PD cases of Germany from 2016 to 2020. NTM incidence and prevalence were calculated for every study year. The presented epidemiological indicators are particularly reliant as TB surveillance data were used as a reference and TB notification reaches almost 100% in Germany. Laboratory incidence and prevalence of NTM recovered from respiratory samples ranged from 4.5–4.9 and from 5.3–5.8/100,000 for the population of Germany, respectively, and did not change over the five-year study period. Prevalence and incidence were stable also when stratifying for facultative pathogenic NTM, *M. avium/intracellulare* complex (MAIC), and *M. abscessus/chelonae* complex (MABSC). The proportion of NTM with drug susceptibility testing (DST) increased from 27.3% (2016) to 43.8% (2020). The unchanging laboratory NTM prevalence/incidence in Germany represents a “ceiling” of possible NTM-PD notification when diagnostic strategies do not change in the coming years. A notable increase in NTM-DST may indicate better notification of NTM-PD and/or awareness of new clinical guidelines but still remains below clinical needs.

## Introduction

Non-tuberculous mycobacteria (NTM) pulmonary disease (PD) is a serious threat to vulnerable patient populations both globally and in Germany [1–4]. The majority of NTM species are environmental commensals or animal pathogens, typically accidentally found in humans, while only few *Mycobacterium species* cause clinical NTM infections. The American Thoracic Society (ATS) and the Infectious Disease Society of America (IDSA) have established diagnostic standards for NTM-PD, according to which three criteria must be met: 1st, microbiological evidence of NTM in respiratory samples, 2nd, the presence of clinical and 3rd, radiological signs consistent with NTM-PD [5]. The vast majority of NTM pulmonary diseases are caused by species of the so-called *Mycobacterium avium-intracellulare* complex (MAIC) [6,7], though less frequent, *M. xenopi*, *M. kansasii*, *M. malmoense*, and *M. abscessus/chelonae* complex (MABSC). Several new taxa are described every year [8] and the number of scientific reports has increased more than 20 times over the last 10 years, potentially indicating that NTM infections are rapidly emerging [9–12].

The overall incidence of NTM-PD worldwide might range somewhere around one to five per 100’000 persons [13] and may be increasing [14–16]. However, it is unclear the extent this is due to a true increase of NTM-PD, or rather their detection and notification are increasing due to better medical awareness of this disease, and improved diagnostic tools [17–19]. When the number of cases with NTM-PD would increase, numbers of NTM isolation in diagnostic laboratories should also rise. There are also conflicting findings, indicating that NTM isolates could be stable or even decreasing [20]. Incidence and prevalence of NTM-PD has been previously estimated for Germany based on statutory health insurance data [3,4]; however, due to the limitations of using such codes it is likely that the prevalence is underestimated. As disease due to NTM are not reported to public health services, the real numbers of prevalent/incident cases are unknown in almost all countries worldwide. However, in Germany all TB cases are reported reliable by both laboratories and clinicians. As the same mycobacteriological laboratories recover both TB species and NTM using the same methods, the reported TB cases can be used as reference for a fairly accurate estimation of prevalence and incidence of NTM isolation in Germany.

Treatment success of NTM disease varies greatly between individual species where some are more easily managed (e.g. *M. kansasii*) than others (e.g. *M. abscessus subsp. abscessus*). Current American [20] and British [21] guidelines strongly recommend to use CLSI standards [22] to test the susceptibility of NTM recovered from patients with pulmonary disease that are believed to be clinical significant before administration of anti-NTM treatment regimens. However, there is no standard method of antibiotic susceptibility testing for NTM [23], and the methods described by CLSI are only performed in some specialized German laboratories after being ordered by clinical doctors [23]. Currently, the proportion of NTM isolates that are tested for drug susceptibility in Germany is not known.

The objectives of the current study were to: (1) estimate the prevalence and incidence of NTM isolation in German microbiological laboratories from 2016 to 2020; (2) determine which NTM species are most commonly detected and if there are regional differences in the NTM species their distribution; and (3) determine the proportion of NTM samples that are tested for antibiotic susceptibility.

## Material and methods

### Study design and laboratory recruitment

This is a retrospective epidemiological study, utilizing data from diagnostic laboratory information management systems (LIMS) across Germany. Participants were affiliated with universities and private medical diagnostic providers and were recruited by phone, email, and an informative print flyer. Collaboration contracts were established primarily to ensure data safety and included a compensation fee of up to 450 EUR for extracting the required data.

All participating laboratories were ISO 15189 accredited and followed the German mycobacteriological diagnostics standards defined by the MIQ 5 Tuberculosis & Mycobacteriosis (Quality standards of microbiological diagnostics of infectious diseases on behalf of the German Society of Hygiene and Medical Microbiology) [24]. Following those guidelines, the laboratories decontaminated all samples with NALC-NaOH, inoculated two solids (e.g. Loewenstein-Jensen and Stonebrinck) and one liquid Middelbook 7H9 culture medium (e.g. MGIT, BD, Heidelberg, or BactALERT, BioMerieux, Nürtingen, Germany), and incubated them for up to eight weeks. Smears for fluorescence microscopy were stained with Auramine O, for bright field microscopy according to Ziehl-Neelsen or modified ZN protocols. At least 300 vision fields were read before a smear was called negative. The adherence to those standards was monitored by internal, external, and yearly audits of the German Accreditation Authority DAkkS.

### Data extraction and cleaning

The participating laboratories extracted LIMS-data for all positive isolates recovered from respiratory samples that grew *Mycobacterium species* (both NTM and MTBC) in culture within the years 2016–2020. Samples were categorized as respiratory when they were sputum, lung tissue, bronchial or alveolar lavage, bronchial or tracheal secretions, or otherwise labelled as pulmonary or respiratory liquid, biopsy or tissue. The primary data extracted for each corresponding isolate included anonymized patient ID, the first three digits of the zip code of the patient’s residence, age, sex, type of sample, location of sample taken from body, identified (sub-) species of mycobacteria, and method of differentiation. When antibiotic susceptibility testing was performed, susceptibility profiles and testing method were included. “Facultative pathogenic” NTM are defined as primarily environmental bacteria that can occasionally cause infection [25], whereas “non-pathogenic” are those which do not, or rarely cause infection. NTM species were categorized into the categories “facultative pathogenic” or “non-pathogenic” by two clinical NTM experts based on their clinical experience and knowledge of the literature (supplementary Table 1). All statistics were performed using R Core Team (2021) [38].

### Calculating the diagnostic coverage of data for Germany

Diagnostics are performed by the same laboratories using identical methods for both TB and NTM. As TB is a notifiable disease, its frequency was used as a reference for the calculation of epidemiological parameters of NTM isolation. NTM isolation prevalence and incidence were extrapolated based on the Diagnostic Coverage calculated with the numbers of incident bacteriologically confirmed active TB cases.

$$\begin{array}{rl} Diagnostic\;Coverage\;in\;Year\;X\\ & =\frac{TB\;incidence\;reported\;by\;laboratories\;in\;Year\;X}{TB\;incidence\;reported\;by\;RKI\;in\;Year\;X}\;\\ & \times 100 \end{array}$$

A TB case was defined as bacteriologically confirmed and active when MTBC grew in the culture from his samples and/or when positive results were obtained in smear microscopy and MTBC PCR. The diagnostic coverage was estimated by dividing the number of incident TB cases detected in the participating laboratories by the number of incident TB cases reported by the German health authority, Robert Koch Institute (RKI).

### Incidence and prevalence

An NTM case (NC) was categorized for the first NTM of each species of a patient’s respiratory samples within a calendar year. By definition, a patient with NTM was considered an NC when NTM grew in the culture from his samples and/or when positive results were obtained in smear microscopy and universal mycobacterium PCR but a negative result in MTBC PCR. In reality, however, positive cultures with NTM were obtained for all cases with positive smear microscopy and mycobacterium PCR so that based on the data NC could be reduced to the positive cultures. If more than one NTM species was isolated from different samples of the same patient, each species was categorized as an NC within that year. Follow-up isolates of the same species in subsequent years would also be categorized as a NCs. This applied method for the NC identification accounts for any change or shift in the species isolated within a single patient to be included in the incidence and prevalence counts for each category but excludes multiple samples of the same species within the same year from the same patient. Mixed cultures (more than one species isolated from a single sample) were included in the prevalence and incidence estimates, where all identified NTM species would be counted as NCs. Incident cases were defined as NCs that had not been identified in the data from years prior. Estimating incident cases in 2016 was not possible as no data for 2015 was received. Prevalence estimates were calculated by counting all NCs for the study period.

Prevalence and incidence were estimated for pulmonary isolates in the following four categories “all NTM”, “MAIC”, “MABSC” and “facultative pathogenic NTM” for all of Germany using the yearly RKI reports for the years 2016–2020 described above [26–30]. The 100% incidence and prevalence for NTM was extrapolated based on the proportion of TB captured by participating laboratories base on the equation below:

$$100\text{\%}\;NTM\;incidence\;(or\;prevalence)\;estimate\;for\;Year\;X=\frac{\begin{array}{rl} & \;NTM\;incidence\;or\;(prevalence)\;\\ & reported\;by\;laboratories\;in\;Year\;X \end{array}}{Diagnostic\;Coverage\;in\;Year\;X}\;$$

The incidence and prevalence per 100,000 population for each year were calculated based on the extrapolated estimate for 100% of the population (above), and the population of Germany for each respective year [31]. Standard 95% confidence intervals were calculated for both incidence and prevalence.

### Proportion of specific NTM species and criteria of NTM-PD

The proportion of the NTM species most commonly isolated in Germany each year (2016–2020) was calculated, with significant differences detected using a Chi^2^ test. Only pure NC cultures with a single NTM species (excluding 67 mixed culture samples) were used for the proportion estimates. Regional proportions of NTM species isolation were calculated, testing for any difference between regions using a Pearson’s chi^2^.

The microbiological diagnostic criteria for NTM-PD are cultures growing NTM from at least two separate expectorated sputum samples; or from at least one bronchial secretion, wash or lavage; or from a transbronchial or other lung biopsies [5]. For each of the top 11 species, and 3 category groups (MAIC, MABSC, *M. fortuitum* group), the proportion of cases fulfilling those criteria were calculated.

### Drug susceptibility testing (DST)

The proportion of drug susceptibility testing (DST) performed for the groups “all NTM”, “facultative pathogenic NTM”, MAIC, MABSC and “non-pathogenic NTM” was calculated for 2016 and 2020 from the total amount of NTM isolated (patient cleaned, i.e. only one isolate of the species counted per patient) from laboratories where DST is performed. Follow-up samples of the same species of patients that may have received NTM therapy were excluded to remove any possible bias that may have been introduced due to antimicrobial resistance in the NTM strain. Additionally, stratification by drug for the most frequently tested drugs was done in order to determine any trends that may have occurred as a result of the updated guidelines in 2018 [21,22,33] and 2020 [34]. Percent change was used to determine magnitude of the change between 2016 and 2020.

## Results

**Figure 1.**
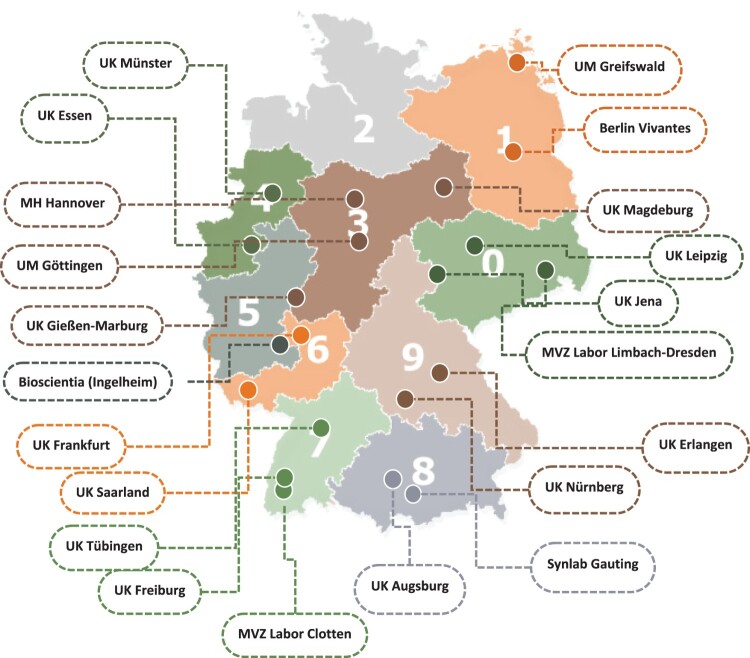
Map of participating laboratories where non-tuberculous mycobacteria (NTM) data for 2016–2020 was received with associated first digit of zipcode. UK = university hospital; UM = university institution; MH = medical school; MVZ = polyclinic care centre.

**Figure 2.**
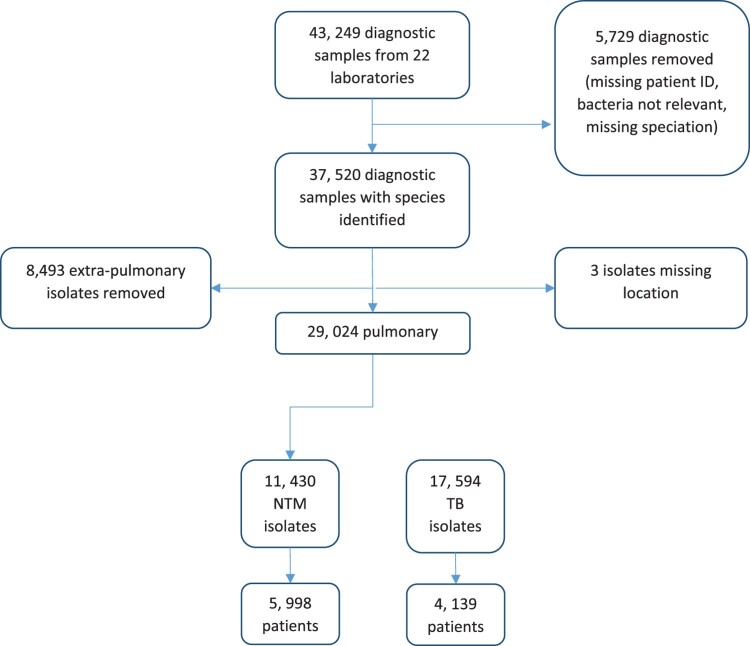
Summary of data received from all participating laboratories and included in the final analysis.

Twenty-two laboratories were recruited as collaborators, sending all diagnostic TB and NTM data from their respective database for the years 2016–2020 (Figure 1). The final database from all participating laboratories contained microbiological information from a total of 43,249 samples. All samples missing patient ID, bacteriological confirmation data of TB or NTMs (*n* = 5729) were excluded from analysis (Figure 2). Only pulmonary samples were included, resulting in 17,594 MTBC isolates from 4139 patients with pulmonary TB identified as the primary isolate, and 11,430 NTM isolates identified from 5998 pulmonary patients (Figure 2). Information regarding negative samples were not submitted from all laboratories, therefore it was not possible to calculate the proportion of samples that were positive in the lab, or on a patient level. Of the positive NTM cases, 55.7% were from male patients (34 missing sex) and the median age was 68 years old (range: 1–100; average 64 years old; 333 missing age). There was a median of two follow-up samples from individual NTM patients in the data set (range: 0–50; IQR: 1, 4), and a median of four follow-up samples for TB patients (range: 0–60; IQR: 2, 8). Follow-up samples from a previously identified species were excluded from the analysis within individual study years.

The laboratories participating in the current study detected 30.7% of all pulmonary TB cases for all 5 years in Germany as reported by the RKI [26–30]. Therefore, we estimated the same coverage for NTM incidence across Germany, as the same diagnostic tests and indicators for testing are used. This resulted in an estimated German prevalence of pulmonary NTM isolates ranging from 5.1 to 5.8/ 100,000 in 2016–2020 (Table 1). For pulmonary facultative pathogenic NTM species (supplementary Table 1), the estimated prevalence ranged from 4.0 to 4.3/100,000 with the incidence ranging from 3.4 to 3.9/100,000 (Table 1). The incidence and prevalence did not change when comparing years 2016–2020 for NTM, facultative pathogenic NTM, MAIC or MABSC.

**Table 1.** German-wide prevalence and incidence estimates for non-tuberculous mycobacteria (NTM) isolated in mycobacteriology laboratories, stratified by “all NTM,” facultative pathogenic NTM (abbreviated to “fac. path”), *Mycobacterium avium*/*intracellulare* complex (“MAIC”), *Mycobacterium abscessus* complex (“MABSC”) and *Mycobacterium tuberculosis* complex (“TB”) for 2016–2020.

Year	Group	Number of incidentpulmonary cases^a^	Number ofprevalentpulmonary cases	Bacteriologically confirmed,incident pulmonary TB casesreported to RKI^b^	Incidence per 100.000population (95% Cl)	Prevalence per 100.000population (95% CI)
2016	All NTM		1265			5.11 (4.96; 5.27)
	fac. path.		990			3.99 (3.86; 4.14)
	MAIC		662			2.67 (2.56; 2.79)
	MABSC		110			0.45 (0.40; 0.49)
	TBC		1031	3283		
2017	All NTM	1243	1349		4.88 (4.73; 5.03)	5.38 (5.22; 5.54)
	fac. path.	986	1089		3.87 (3.73; 4.0)	4.34 (4.20; 4.49)
	MAIC	665	726		2.61 (2.5; 2.72)	2.89 (2.78; 3.01)
	MABSC	86	115		0.34 (0.3; 0.38)	0.46 (0.41; 0.51)
	TB	966	1007	3077		
2018	All NTM	1196	1323		4.79 (4.64; 4.94)	5.49 (5.34; 5.65)
	fac. path.	886	1008		3.55 (3.42; 3.68)	4.18 (4.05; 4.33)
	MAIC	585	665		2.34 (2.24; 2.45)	2.76 (2.65; 2.88)
	MABSC	87	111		0.35 (0.31; 0.39)	0.46 (0.42; 0.51)
	TB	963	993	3140		
2019	All NTM	1226	1379		4.88 (4.73; 5.03)	5.75 (5.59; 5.92)
	fac. path.	904	1044		3.6 (3.47; 3.73)	4.36 (4.22; 4.50)
	MAIC	623	710		2.48 (2.38; 2.59)	2.96 (2.85; 3.08)
	MABSC	80	112		0.32 (0.28; 0.36)	0.47 (0.42;0.52)
	TB	865	891	2795		
2020	All NTM	1132	1284		4.51 (4.36; 4.65)	5.32 (5.17; 5.48)
	fac. path.	863	992		3.44 (3.31; 3.57)	4.14 (3.98; 4.25)
	MAIC	589	680		2.35 (2.24; 2.45)	2.82 (2.70; 2.94)
	MABSC	86	113		0.34 (0.3; 0.39)	0.47 (0.42; 0.52)
	TB	744	766	2461		

^a^ Incidence not calculated for 2016 as the previous year’s data not available.

^b^ Robert Koch Institute (RKI) yearly TB reports [26–30].

**Figure 3.**
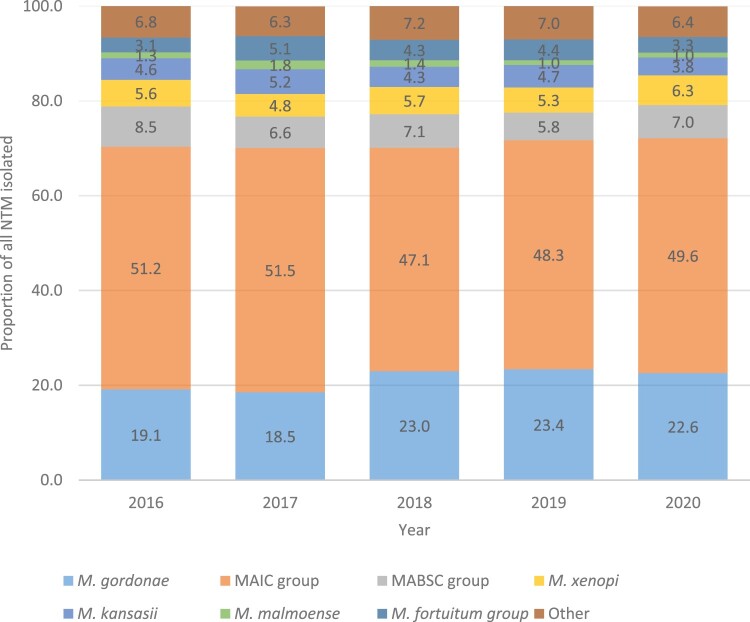
Proportion of non-tuberculous mycobacteria (NTM) isolated in participating mycobacteriology laboratories for 2016–2020. Proportions include only pure cultures with a single NTM isolate, and do not include follow-up samples collected within the same year.

The most commonly isolated NTM species were from the MAIC complex of all NTM isolated accounting for 49.6% for all 5 years, followed by *M. gordonae* (21.3%; Figure 3). MABSC isolates accounted for 7.0% of all NTM isolates identified in microbiological laboratories. No difference in the 5-year period for proportion of any of the facultative pathogenic stratified species was observed. However, *M. gordonae* proportions during the 5-year period was significantly different (*p* = 0.006), with greatest difference occurring in 2017 (*p* = 0.01).

All years were combined to analyse if any regional differences existed for the eight most frequent stratified complexes and species (MAIC, MABSC, *M. fortuitum* group*, M. gordonae M. xenopi, M. kansasii, M. malmoense* and “other”). The “other” category included 75 NTM species that were identified in laboratories, consisting of 452 (6.7%) of all NC NTM isolates. *M. kansasii* was the only species which had no significant difference detected in the proportion isolated between all 10 zipcodes in Germany (Table 2). All other species were isolated in significantly different proportions for all regions with MAIC being isolated most frequently, followed by *M. gordonae* (Table 1). *M. xenopi* was isolated in higher proportions than MABSC in southern regions with zipcodes starting with 7, 8 and 9 (Table 2). The highest proportion of NTM NC came from the Western region with zip code 4 (17.2%), the lowest from the Northern region with zipcode 2 (1.5%) (Table 2).

**Table 2.** Species-specific proportions of non-tuberculous mycobacteria (NTM) that was isolated from pulmonary specimens within each zipcode region for all 5 years (2016–2020). Green highlighting indicates regions where the species is found in higher proportions than column to the left. Red highlighting indicates regions where species is found in higher proportions than MABSC. Proportions include only pure samples with a single NTM isolate is cultured, and do not include follow-up samples collected within the same year. MAIC includes species of the *Mycobacterium avium-intracellulare* complex, MABSC includes those of *M. abscessus/chelonae* complex. *P*-values calculated with Chi².

First digit of zipcode	MAIC*	M. gordonae*	MABSC^†^	*M. Kansasii*	*M. fortuitum* Group*	M. xenopi*	M. malmoense*	Other*	Regional proportion of total NTM isolated*
0	48.5%	20.2%	6.9%	4.6%	3.3%	4.3%	3.3%	8.9%	12.3%
1	47.0%	22.5%	5.5%	3.9%	7.4%	2.9%	1.1%	9.7%	8.2%
2	48.9%	21.1%	10.0%	2.2%	4.4%	1.1%	2.2%	10.0%	1.5%
3	47.3%	23.5%	7.9%	4.6%	5.2%	2.9%	1.2%	7.4%	10.0%
4	61.6%	15.8%	8.0%	4.5%	2.4%	2.8%	0.8%	4.2%	17.2%
5	59.9%	19.2%	4.1%	3.3%	2.8%	4.2%	1.7%	4.9%	13.6%
6	46.2%	25.5%	7.7%	4.2%	4.9%	4.5%	1.1%	6.0%	13.4%
7	42.0%	23.3%	7.6%	5.8%	3.8%	11.1%	0.5%	5.8%	9.8%
8	43.8%	14.5%	9.0%	5.5%	5.9%	9.6%	2.0%	10.0%	8.4%
9	43.8%	25.3%	7.4%	4.0%	3.4%	7.7%	0.9%	7.4%	5.7%

*Significant difference between regions (*p* < 0.001).

^†^ Significant difference between regions (*p* < 0.05).

The category MAIC had the highest number patients, followed by *M. gordonae* and MABSC (Table 3). One laboratory did not submit follow-up data and was therefore excluded from follow-up results. There were 937 (15.%) patients that had at least two samples collected (follow-up samples) of the same species. The species with the highest proportion follow-up samples of the same species from patients were *M. massiliense* (66.7%), *M. abscessus* (47.1%) and *M. malmoense* (40.0%) (Table 3). A larger proportion of patients met the BAL/surgical criteria of the ATS/IDSA for NTM pulmonary disease than the follow-up sample criteria for species in the MAIC, *M. malmoense*, *M. kansasii*, *M. xenopi*, and *M. gordonae* (Table 3).

**Table 3.** The number of pulmonary samples and patients with non-tuberculous mycobacteria (NTM) isolation for the indicated complex/species from mycobacteriological laboratories with follow-up data in 2016–2020. The number and proportion of patients meeting microbiological criteria of ATS/IDSA [5] for NTM pulmonary disease are represented of all patients with at least one laboratory-confirmed case of NTM; (1) Positive culture from two separate samples and (2) samples collected from bronchial wash or lavage (BAL) or surgical biopsy.

Species	Number of positive pulmonary samples	Number of patients	Number of patients with subsequent samples with the same species (% of patients)	Number of patients with samples collected from BAL or surgical biopsy (% of patients)
MAIC^a^	6128	3082	600 (19.5)	1697 (55.1)
* M. avium*	2689	1237	299 (24.2)	752 (60.8)
* M. intracellulare*	1652	832	169 (20.3)	392 (47.1)
* M. chimaera*	821	489	59 (12.1)	295 (60.3)
MABSC^b^	1230	432	111 (25.7)	106 (24.5)
* M. abscessus*	689	172	81 (47.1)	54 (31.4)
* M. bolletii*	70	20	7 (35.0)	5 (25.0)
* M. massiliense*	119	21	14 (66.7)	12 (17.8)
* M. chelonae*	141	118	4 (3.4)	21 (17.8)
* M. malmoense*	243	85	34 (40.0)	52 (61.2)
* M. kansasii*	602	288	75 (26.0)	173 (60.1)
* M. xenopi*	515	342	42 (12.3)	206 (60.2)
* M. fortuitum* group	295	244	8 (3.3)	51 (20.9)
* M. gordonae*	1528	1342	33 (2.5)	572 (42.6)

^a^ Any NTM identified as *Mycobacterium avium/intracellulare* complex.

^b^ Any NTM identified as *Mycobacterium abscessus/chelonae* complex.

**Figure 4.**
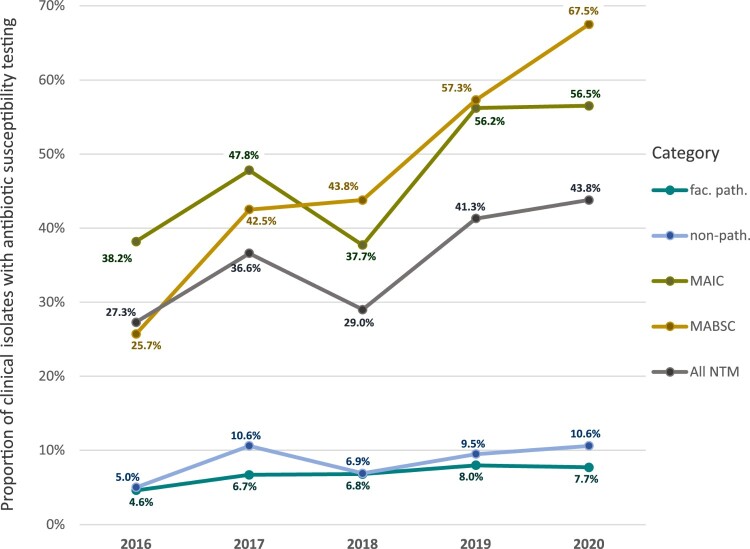
Proportion of “all NTM”, “MAIC”, “MABSC”, “facultative pathogenic (fac. path.)” and “non-pathogenic (non-path.)” that had drug susceptibility testing (patient-cleaned). “Facultative pathogenic NTM” group does not contain MAIC and MABSC, as those are displayed separately. *DST data from one laboratory not available in 2018.

**Figure 5.**
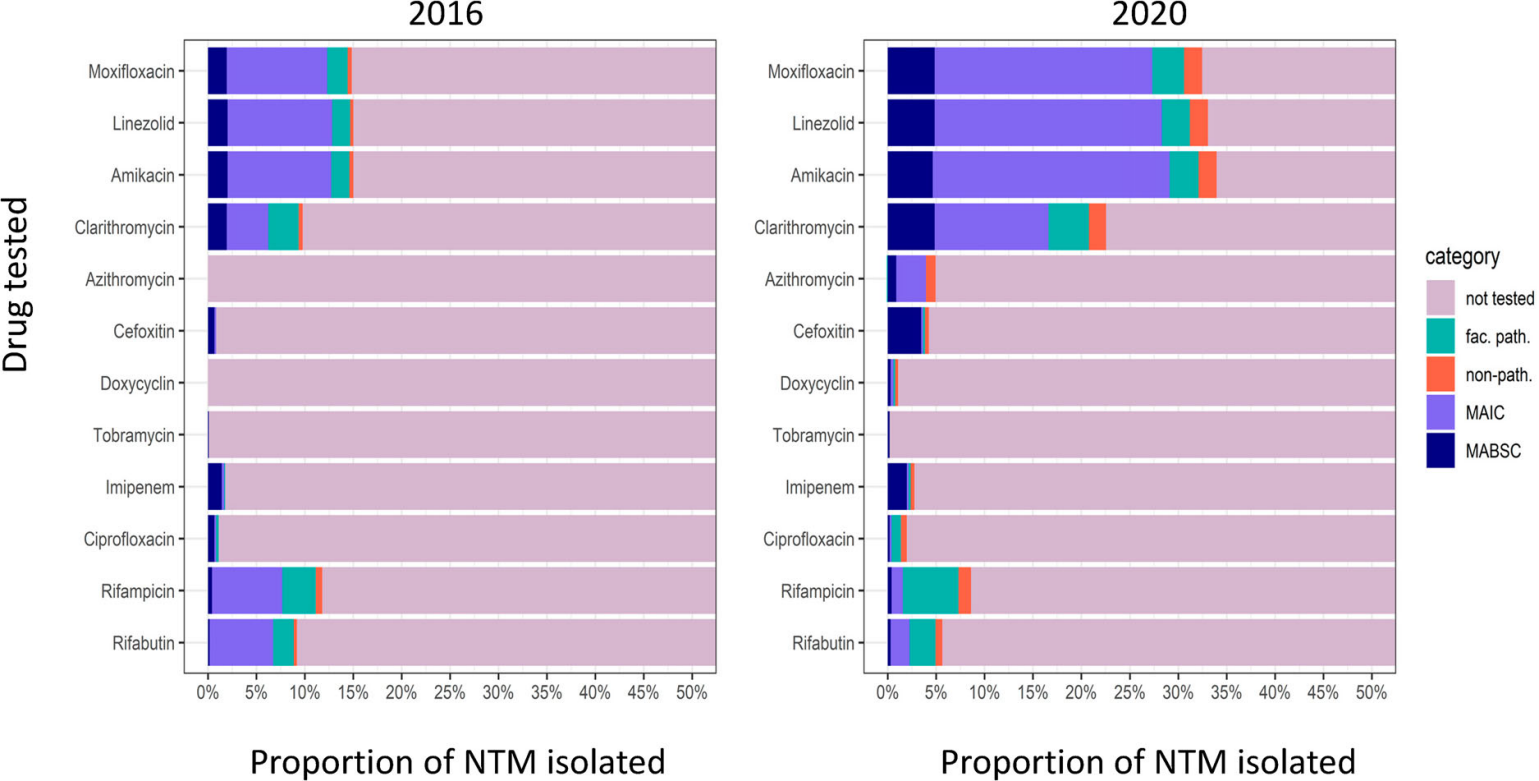
Proportion of non-tuberculous mycobacteria (NTM) isolates that were tested for specific drugs, stratified by “facultative pathogenic” NTM (abbreviated to “fac. path”; not including MAIC and MABSC), MAIC, MABSC and “non-pathogenic” NTM (abbreviated to “non-path”). The total number of isolates (patient cleaned) from laboratories (*n* = 17) that have performed NTM-DST in 2016 and 2020 were 320 and 436, respectively.

Over the 5-year study period (2016–2020), the proportion of NTM isolates tested in laboratories where DST is conducted (*n* = 17 institutions) increased from 27.3% to 43.8% for all drugs, with the greatest increase observed for MAIC (Figure 4). There was an artificial decrease in the testing of MAIC in 2018 (Figure 4), but should not be considered relevant as DST data from one laboratory accounting for about 35% of MAIC samples tested each year was unavailable in 2018. In 2016, the most frequently tested drugs were Moxifloxacin (MXF), Linezolid (LZD), Amikacin (AMK) and Rifampicin (RIF; Figure 5). In 2020, there was an increase in testing for MXF, LZD, AMK, and Clarithromycin (CLA) for all groups, with these four drugs being tested in the highest proportions (MXF = 32; LZD = 33%; AMK = 34%; and CLA = 23%; Figure 5) even for non-pathogenic NTM. Testing with Azithromycin increased from 0% of isolates in 2016 to 5% of isolates in 2020, with the greatest increase in MAIC and non-pathogenic NTM (Figure 5). There was a large decrease in the number of MAIC being tested for Rifabutin and RIF (5% decrease, 79% change and 6% decrease, 84% change; respectively); however, testing of these drugs increased in other pathogenic (27% change and 66% change; respectively) as well as non-pathogenic NTM (105% change and 90% change; respectively).

## Discussion

Data on NTM incidence and prevalence in Central Europe are scarce. In this study, the prevalence of NTM isolated in German mycobacteriological laboratories was estimated to range between 5.1 and 5.6/100,000 from 2016 to 2020. The prevalence did not change over the 5-year study period, and may represent a “ceiling” of possible NTM-PD diagnosis in the current, and upcoming years. A notable increase in DST performed on NTM isolated in the current study, may indicate a better awareness of international NTM guidelines [5,34] and potentially improved notification of NTM-PD among all patients with NTM in their respiratory tract. We found some regional particularities in the NTM species distribution, with *M. xenopi* being isolated far more frequently in the southern regions.

The incidence of patients with facultative pathogenic NTM recovered from their pulmonary samples ranged between 3.6–3.9/100, 000 in the years 2016–2020, higher than the overall incidence and prevalence estimated in previous studies conducted in 2010 (incidence NTM-PD: 2.6/100,000) [3] and from 2009–2014 (prevalence NTM-PD rising from 2.3 to 3.3/100,000) [4]. While the current study was based on mycobacteriological laboratory data, the cited past studies relied on clinical diagnosis of NTM-PD coded with the diagnosis-related groups (DRG) for the statutory health insurance [3,4]. The number of NTM-PD diagnoses is *per se* lower than numbers of NTMs recovered from respiratory specimens since a significant proportion of NTMs occur only as commensals without clinical relevance. Furthermore, DRG coding has a risk of underreporting since the value of the German NTM-PD DRG does even not cover the full costs of nursing the patient in a hospital. NTM isolation prevalence in the laboratories was not found to be increasing over 5 years; however, there may still be an underestimation and underdiagnosis of NTM-PD [31] and focus should be on early detection to better treat patients.

The diagnostic NTM-PD criteria of the ATS require 1st the evidence of NTM in samples from the respiratory tract, 2nd clinical, and 3rd radiological signs consistent with pulmonary mycobacteriosis [5]. Numbers of NTM-PD can thus not exceed the number of NTMs detected by mycobacteriological labs. The real number of NTM-PD could be theoretically higher than the number of cases with NTM found in the labs when a large amount of NTM-PD remained undiagnosed. This is however unlikely for the years investigated. As a result of the particularly high wave of refugees that reached Germany in 2015, the incidence of TB shot up 1.4 times [26,32]. This sharpened the awareness of German doctors for TB reflected also by the high numbers of mycobacteriological orders sent to laboratories until the outbreak of COVID-19 in Germany (own unpublished data). Since NTMs are predominantly found in the course of tuberculosis diagnostics, a relevant underdiagnosis of NTM-PD seems thus unlikely. We would therefore consider the incidence / prevalence rates found in our study as ceiling values that will most likely not be exceeded by NTM-PD in the years to come.

Similar to our findings, Henkle et al. (2015) reported a stable incidence (5/100,000) that did not significantly increase over a six-year period from 2007 to 2012 [39] in Oregon, US. The authors used similar methods to our study though not including the reference of notifiable TB epidemiology. However, increase or stability of NTM incidence/prevalence may be region specific as previous studies in Canada [20] and South Korea [15] reported significant increase in NTM that is being isolated from clinical samples in mycobacteriological laboratories. Recent review literature reported significant differences between regions and countries for the rates of pulmonary NTM isolation and disease, the population at risk, and the risk factors for disease development [40].

The most commonly isolated species in this study (MAIC [49.6%], *M. gordonae* [21.3%] and MABSC [7.0%]), are also the most commonly observed NTM around the world [15,35,40,41]; except in Canada [19], where *M. xenopi* is more frequently isolated. A higher endemicity for *M. xenopi* has already been reported for different countries and regions within countries, such as the Ostrava district in the Czech Republic [42], Hungary, Croatia, Belgium, South-East England, Northern France or the Barcelona area in Spain [37]. The NTM species distribution appears to be geographically different around the world [37], but also within Germany based on our current findings. The proportion of specific species being isolated within regions was found to be significantly different for all regions, therefore, the epidemiological research of NTM-PD should shift towards species-specific distribution and prevalence rates when determining geographical and temporal trends.

In 2016, the German Central Committee against Tuberculosis published the national policy that DST of NTM should not be routinely performed due to the limited number of clinical evaluations for *in vitro* and *in vivo* correlation and DST methods [23]. In 2018, new guidelines of CLSI and the British Thoracic Society were published strongly promoting DST of NTM at least for those drugs that had sufficient scientific evidence of *in vitro* – *in vivo* treatment response correlation [21,22,33]. It is likely that due to new recommendations, DST increased in laboratories where DST testing was conducted, indicating a general shift towards treatment informed by susceptibility testing in Germany [5,21,22]. Nevertheless, the overall proportion of tested NTM in Germany still remains low.

The trend towards more DST of MAIC for clarithromycin and amikacin resistance along with the decreased testing of rifampin and rifabutin are in line with the CSLI guidelines [22,33] and British Thoracic Society guidelines [21]. It appears that the new guidelines in 2018 had an effect on the overall proportion of testing conducted in Germany, as well as directing which drugs to be tested based on the species of NTM that is isolated. As there have been no recent recommendations published in Germany for DST of NTM, there may still be a lack of clear understanding among clinicians whether susceptibility testing of NTM is necessary. However, it needs to be considered that susceptibility testing is only recommended for patients receiving treatment [5]; therefore, the apparent low proportion of testing may be due to a lack of decision to treat which is in contrast to the current NTM-PD treatment guidelines that suggest initiation of treatment rather than watchful waiting [5].

There are limitations to the current study that should be considered. As no negative samples were included in the data collection or analysis, all proportions are calculated from positive samples identified in the participating laboratories. Future studies should consider collecting data on negative samples as well so that the recovery rate of NTM from all laboratory samples can be estimated. However, this is the first time representative population-based estimates of NTM isolation in Germany, which is based on isolation in mycobacteriology laboratories of this magnitude, could be made. This was possible as we were able to combine comprehensive data from the information management systems of participating laboratories with health service records of the RKI. As only laboratory data was collected, the presented incidence and prevalence rates only refer to laboratory isolates, not to NTM-PD. Although a substantial proportion of cases fulfilled the microbiological ATS criteria of NTM-PD, it remains unknown how many of those also fulfilled the clinical and radiological criteria [5]. Unfortunately, not all contacted laboratories participated in the study; therefore, estimates are based only on those that submitted data. However, as we were able to include more than 30% of all TB cases that had been bacteriologically confirmed in Germany during the study period, we would consider our data set highly representative. Finally, because not all laboratories submitted data on DST, proportion estimates were based only on positive samples from laboratories where that data was available.

In summary, the population incidence and prevalence for all NTM, facultative pathogenic NTM, MAIC and MABSC were estimated for the German population based on the coverage of data obtained, determined by TB diagnosis reported to the German health authority (RKI). The higher prevalence and incidence estimates in the current study compared to previous studies may be a better representation of NTM isolation (rather than NTM-PD) in the country due to this adjustment. There appears to be an increase from 2016 to 2020 in the amount of DST that is being conducted for NTM isolates, with a change in the specific drugs being tested, seemingly in accordance with the 2018 CLSI guildelines [33] suggesting an increase of notification or even in clinical relevance of facultative pathogenic NTM species. Future studies should investigate the potential impact of intensified testing on NTM notification, the proportion of patients with NTM in respiratory samples that are finally diagnosed with NTM-PD and put on treatment, applied treatment regimens, case management, monitoring and clinical outcomes.

**List of abbreviations**
AMK: Amikacin
ATS/IDSA: American Thoracis Society/Infectious Diseases Society of America
CLA: Clarithromycin
CLSI: Clinical and Laboratory Standards Institute
DST: Drug susceptibility testing
LZD: Linezolid
MTBC: *Mycobacterium tuberculosis complex*
MTB: *Mycobacterium tuberculosis*
MABSC: *Mycobacterium abscessus/chelonae* complex
MAIC: *Mycobacterium avium/intracellulare* complex
MXF: Moxifloxacin
NTM: Non-tuberculous mycobacteria
NC: NTM case
NTM-PD: Non-tuberculous mycobacteria pulmonary disease
RIF: Rifampicin
TB: Tuberculosis
RKI: Robert Koch Institute

## Ethics approval and consent to participate

Permission to conduct the study was obtained from the Ethical Committee of the Ludwig-Maximilian’s University, Munich, Germany under project number 20-0126.

## Supplementary Material

Supplementary_Table

## Data Availability

The datasets used and/or analysed during the current study are available from the corresponding author on reasonable request.
