# Supplementary material for: Development of prevalence and incidence of non-tuberculous mycobacteria in German laboratories from 2016 to 2020
Source: Emerg Microbes Infect. 2023 Dec 6;12(2):2276342. doi: 10.1080/22221751.2023.2276342 (PMC10769520; doi:10.1080/22221751.2023.2276342)
Supplement: Supplementary_Table [file TEMI_A_2276342_SM3989.docx]

Supplementary Material

**Supplementary Table 1:** Categorization of “facultative pathogenic”, “non-pathogenic NTM species”, MAIC and MABSC by two clinical non-tuberculous mycobacteria (NTM) experts.

| **Category** | **Species name** | | | | | | |
| --- | --- | --- | --- | --- | --- | --- | --- |
| *Mycobacterium avium/intracellulare* complex (MAIC) | *M. avium*  *M. intracellulare*  *M. chimaera* |  |  | | |  | |
|  |  |  |  | | |  | |
| *Mycobacterium abscessus c*omplex (MABSC) | *M. abscessus*  *M. bolletii*  *M. chelonae*  *M. massiliense* |  |  | | |  | |
|  |  |  |  | | |  | |
| Facultatively Pathogenic NTM |  |  |  | | |  | |
|  | MAIC | *M. fortuitum* | *M. lentiflavum* | | | *M. septicum* | |
|  | MABSC | *M. fortuitum group* | *M. mageritense* | | | *M. shimoidei* | |
|  | *M. abscessus* | *M. gastri* | *M. malmoense* | | | *M. simiae* | |
|  | *M. arosiense* | *M. genavense* | *M. marseillense* | | | *M. simiae group* | |
|  | *M. avium* | *M. goodii* | *M. massiliense* | | | *M. szulgai* | |
|  | *M. avium spp. Paratuberculosis* | *M. haemophilum* | *M. nebraskense* | | | *M. tilburgii* | |
|  | *M. basiliense* | *M. hassiacum* | *M. neoaurum* | | | *M. timonense* | |
|  | *M. bolletii* | *M. heckeshornense* | *M. paraffinicum* | | | *M. triplex* | |
|  | *M. bouchedurhonense* | *M. holsaticum* | *M. parascrofulaceum* | | | *M. vulneris* | |
|  | *M. branderi* | *M. interjectum* | *M. peregrinum* | | | *M. xenopi* | |
|  | *M. celatum* | *M. intermedium* | *M. porcinum* | | | *M. yongonense* | |
|  | *M. chelonae* | *M. intracellulare* | *M. saskatchewanense* | | |  | |
|  | *M. chimaera* | *M. kansasii* | *M. scrofulaceum* | | |  | |
|  | *M. colombiense* | *M. kumamotonense* | | *M. senegalense* |  | |  |
|  | *M. conspicum* | *M. kyorinense* | *M. seoulense* | | |  | |
|  |  |  |  | | |  | |
| Non-pathogenic NTM |  |  |  | | |  | |
|  | *M. agri* | *M. frederikbergense* | *M. mucogenicum group* | | | *M. smegmatis* | |
|  | *M. anyangense* | *M. flavescens* | *M. neglectum* | | | *M. smegmatis group* | |
|  | *M. arabiense* | *M. florentinum* | *M. nonchromogenicum* | | | M. sp.* | |
|  | *M. arupense* | *M. fluoranthenivorans* | *M. noviomagense* | | | M. sp. (NTM)* | |
|  | *M. asiaticum* | *M. gordonae* | *M. novocastrense* | | | *M. terrae complex* | |
|  | *M. aubagnense* | *M. graecum* | *M. palustre* | | | *M. triplex group* | |
|  | *M. bohemicum* | *M. heraklionense* | *M. paraense* | | | *M. triviale* | |
|  | *M. branderi* | *M. hiberniae* | *M. paragordonae* | | | *M. tusciae* | |
|  | *M. celatum* | *M. iranicum* | *M. paraterrae* | | |  | |
|  | *M. confluentis* | *M. kubicae* | *M. phlei* | | |  | |
|  | *M. cosmeticum* | *M. marinum* | *M. pseudokansasii* | | |  | |
|  | *M. dierndorferi* | *M. monacense* | *M. pulveris* | | |  | |
|  | *M. elephantis* | *M. moriokaense* | *M. salmoniphilum* | | |  | |
|  | *M. europaeum* | *M. mucogenicum* | *M. sediminis* | | |  | |

*NTM species not differentiated any further
